# New distribution record of the rare bat *Hypsugo* cf. *vordermanni* (Chiroptera, Vespertilionidae) from the Crocker Range Park, Sabah, Malaysian Borneo

**DOI:** 10.3897/BDJ.10.e87860

**Published:** 2022-09-27

**Authors:** Noor Haliza Hasan, Ummu Safiyyah Daud, Amirrah Amat, Yen Chi Lok, Mohd Farhan Mohd Johar, Juannis Gompoyo, Yit Tu Fred Tuh

**Affiliations:** 1 Institute for Tropical Biology and Conservation, Universiti Malaysia Sabah, Kota Kinabalu, Malaysia Institute for Tropical Biology and Conservation, Universiti Malaysia Sabah Kota Kinabalu Malaysia; 2 Department of Zoology, Sabah Parks, Kota Kinabalu, Malaysia Department of Zoology, Sabah Parks Kota Kinabalu Malaysia

**Keywords:** white-winged pipistrelle, Mantailang

## Abstract

A female Hypsugocf.vordermanni was caught at a stream near a village road in Mantailang, Crocker Range Park, Sabah, on 12 November 2018. This bat is a new record for the national park and the second record for Sabah. It was first recorded from Banggi Island, Kudat, in 1991. The species' few records throughout its range is most likely due to sampling effort bias towards forest interior and cave-dwelling species, as this species is more likely an edge-space aerial forager. It is morphologically similar to Peninsular Malaysia's *Hypsugomacrotis*, but the latter has never been reported from Borneo. Therefore, additional specimen collection and molecular data for H.cf.vordermanni are needed for further species confirmation. More information on *H.vordermanni* ecology is also crucial in aiding the management plan for this species as it is currently classified as Data Deficient by the IUCN.

## Introduction

The vespertiolinid bats (family Vespertilionidae) are the most widespread bats worldwide (approximately 300 species) with 41 species from 14 genera occurring on Borneo Island, namely *Arielulus*, *Falsistrellus*, *Glischropus*, *Hesperoptenus*, *Myotis*, *Philetor*, *Pipistrellus*, *Scotophilus*, *Tylonycteris*, *Murina*, *Kerivoula*, *Harpiocephalus*, *Phoniscus* and *Hypsugo* (Phillipps and Phillipps 2018).

*Hypsugovordermanni*, the white-winged pipistrelle was first described as *Vesperugovordermanni* (Jentink, 1890c) from Belitung Island, Indonesia. It is a small-sized bat with a forearm recorded at 30.5 mm and a tail of 15.1 mm. It is a rare bat with only six specimens recorded from Samunsam, Sarawak (Payne et al. 1985, Francis and Hill 1986), Tanjung Puting, Kalimantan (Nash and Nash 1987), Belitung Island, Indonesia (Corbet and Hill 1992, Lim et al. 2016), Belalong, Brunei (Cranbrook and Edwards 1994), Banggi Island, Sabah (Nor 1996), and Kuching, Sarawak (Abdullah et al. 2000, Abdullah et al. 2010).

Until recently, it was included within *Pipistrellus*, from which *Hypsugo* differs in terms of its skull characteristics: smaller anterior premolar, shorter rostrum, smaller incisor and canine, with a myotodont pattern of the lower molars and broad and short baculum (Horáček and Hanák 1986, Francis 2008). *H.vordermanni* is described as having pale reddish-brown fur with darker bases, with large ears and a slightly hatchet-shaped tragus. The most prominent characteristic of this species is its translucent white wings, which become greyer near its body. Its anterior upper premolar is tiny and displaced inwards, which results in the canine and second premolar touching. It is similar to the Least Pipistrelle, *P.tenuis*, which has darker wings and a narrower tragus (Payne et al. 1985). *H.vordermanni* is also morphologically similar to *H.macrotis*, albeit the latter being relatively larger (Lim et al. 2016). It is noted that *H.vordermanni* feeds on insects and fish in rivers and calm seas (Phillipps and Phillipps 2018).

## Materials and Methods

**Figure 1.**
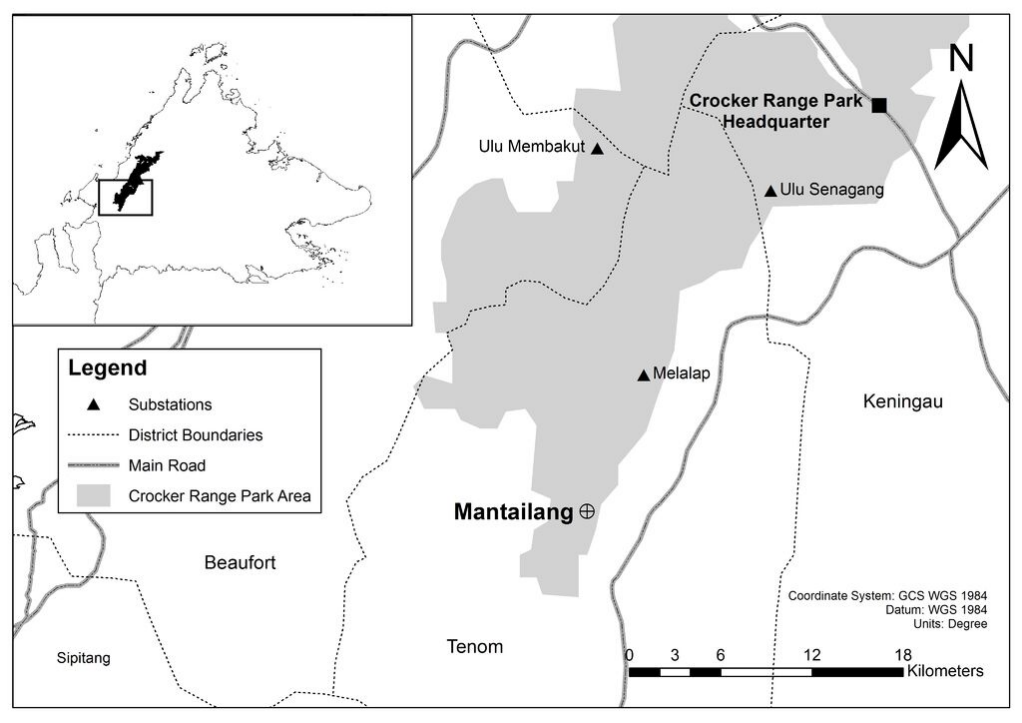
A map showing the location of Mantailang in Sabah, northeast Borneo. Mantailang is an area located near a village road within the border of the Crocker Range Park, a conservation area governed by the Sabah Parks in Sabah, Malaysian Borneo.

**Figure 2.**
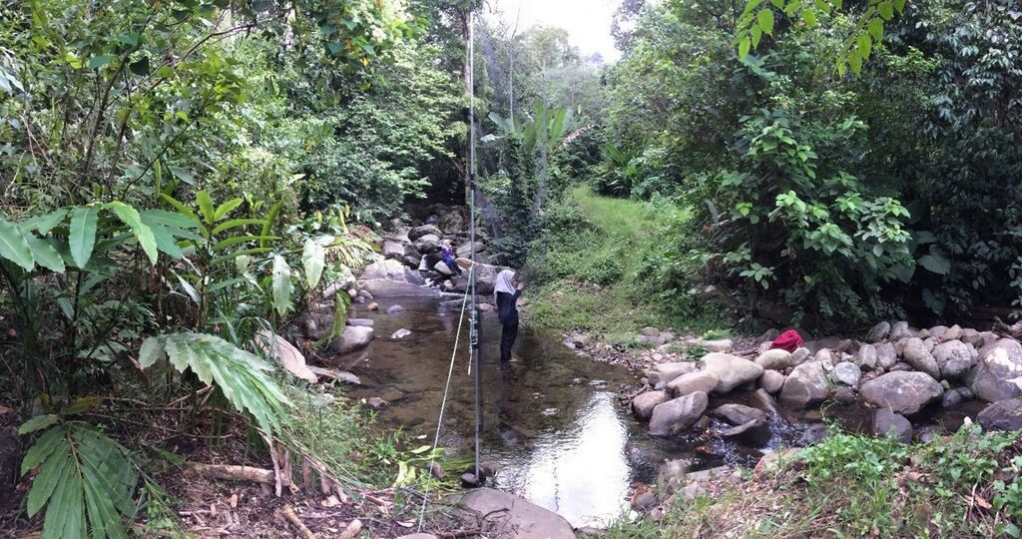
A mist net was deployed across a river stream at the forest edge where the specimen was caught.

On 12 November 2018, a specimen of H.cf.vordermanni was caught in the mist net deployed across a stream near the village road in Mantailang, Crocker Range Park, Sabah, located at 5°10'15.13"N, 115°56 4.41"W (Fig. 1 and Fig. 2). The specimen was measured for its external characteristics, including: forearm length (FA), ear length (E), tragus length (TR), tibia length (TB), hind-foot length (HF), tail ventral length (TVL) and weight (Wt). Measurements were taken using the Mitutoyo Digital Caliper 500-196-30 (Mitutoyo, Japan) and weights were taken using a Pesola spring balance (Pesola AG, Sweden). Identification was done, based on keys in Payne et al. (1985).

## Results and Discussion

**Figure 3.**
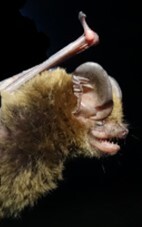
Side profile of H.cf.vordermanni from Mantailang, Crocker Range Park, Sabah, Malaysian-Borneo. The hatchet-shaped tragus is one of the indicative features of this genus.

This is a new distributional record for H.cf.vordermanni in Sabah, Malaysian Borneo. This specimen is an adult, pregnant female, with external measurements of FA 33.58 mm, E 10.1 mm, TR 4.91 mm, TB 15.19 mm, HF 8.34 mm, TVL 35.68 mm and Wt 5.8 g (Fig. 3). It is a poorly known and rare species due to its very low capture rate and is categorised as Data Deficient by the IUCN (Görföl et al. 2016). The key characters which suggest specimen identification as H.cf.vordermanni is the translucent white wings and the hatchet-shaped tragus.

In Asia, only five species of Vespertilionidae bats are characterised by their "whitish' wings ("almost translucent" and "semi-translucent" included), including *Hypsugovordermanni*, *H.macrotis*, *Kerivoulapellucida*, *K.hardwickii* and *Myotismacrotarsus* (Rydell et al. 2020). The *Kerivoula* spp. are easily identified by their woolly fur, funnel-shapped ears, with long and pointed tragus. Meanwhile the *Myotis* spp. have shorter fur and forward-bent tragus (Phillipps and Phillipps 2018).

**Figure 4.**
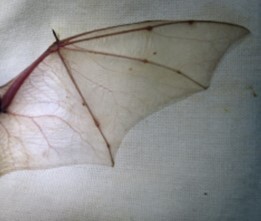
The white translucent wing of *H.vordermanni* against a white cloth as a background.

Hill (1983) and Francis and Hill (1986) remarked that *H.vordermanni* may be conspecific with *H.macrotis*, although the possibility of these two being distinct species was also acknowledged (Corbet and Hill 1992). It is noted that both *H.macrotis* and *H.vordermanni* have large ears and translucent wings (Fig. 4), with *H.macrotis* being relatively larger in size (Francis 2008).

Translucent or whitish wings are suggested to function as camouflage against lit background, thus indicative of the species being an open space aerial forager (Rydell et al. 2020). *H.macrotis* is suggested to be an edge species (Lim et al. 2016), while the specimen described here was caught on the stream located in an open area at the forest edge near a village road.

A previous study recorded *H.macrotis* to have a forearm (FA) range of between 31.7 and 34.5 mm (n = 14), while *H.vordermanni* FA ranged from 31.0 to 33.4 (n = 2) (Lim et al. 2016). A recent study showed that cranial and craniodental characteristics between these two species slightly differ, where *H.macrotis* has a longer skull in comparison to *H.vordermanni* (Lim et al. 2016). The specimen described here showed a FA measurement exceeding the range for *H.vordermanni* by 0.18 mm and overlapped with the FA measurement for *H.macrotis*. However, the FA measurement range for *H.vordermanni* is based on only six (6) specimens (Table 1) and this is not comprehensive enough to represent the whole species. However, it is noted that additional specimen collection and retrieval of molecular data with detailed information on its ecology is a priority for confirming the species identification. Nevertheless, as of today, there is no known record of *H.vordermanni* from Peninsular Malaysia and vice versa for *H.macrotis* from Borneo. Therefore, it is concluded that the specimen recorded in this study is H.cf.vordermanni.

It is a limitation for the current study that no skull measurement is available for the individual captured as it was released as it was a pregnant individual. No molecular data are available for the individual described here, thus limiting the verification options. Nevertheless, findings from this study contributed to additional distribution data of *H.vordermanni* in Sabah. More sampling efforts targeting the streams or water bodies near the forest edge or open areas are suggested to obtain more data on this rare or elusive species.

## Figures and Tables

**Table 1.** Detailed external character measurements for six (6) *Hypsugovordermanni* specimens recorded from Borneo, with the specimen found in this study, are presented. MZU/M= UNIMAS Museum Number; BMNH= British Museum (Natural History); FMNH= Field Museum of Natural History (Chicago); MCR= Mantailang Crocker Range; FA= Forearm; E= Ear; TB= Tibia; HF= Hind-foot; TVL= Tail ventral length; Wt=weight.

**Locality**	**Specimen ID**	**n**	**FA**	**E**	**TB**	**HF**	**TVL**	**Wt**	**Sex**	**Reference**
Samunsam Wildlife Sanctuary, Sarawak, Malaysian Borneo; Belitung Island, Indonesia	BM(NH) 82.547; RMNH 35570 (holotype)	2	31.0 - 33.4	-	-	-	-	-	-	Lim et al. (2016)
Bako National Park, Kuching, Sarawak	MZU/M/01460	1	32.4	8.9	15.7	5.0	14.6	4.5	M	Abdullah et al. (2010)
Annah Rais Penrissen, Kuching, Sarawak	MZU/M/00126	1	33.0	13.3	16.8	-	15.0	5.0	M	
Samunsam, Sarawak	BMNH 82.547	1	30.5	-	-	-	-	-	F	Francis and Hill (1986)
Banggi Island, Sabah	1 FMNH	1	33.0	-	-	-	33.0	6.0	M	Nor (1996)
Mantailang, Crocker Range Park, Tenom, Sabah	MCR051	1	33.6	10.1	15.2	8.3	35.7	5.8	F	Current study
